# Artificial Intelligence (AI) and Nuclear Features from the Fine Needle Aspirated (FNA) Tissue Samples to Recognize Breast Cancer

**DOI:** 10.3390/jimaging10080201

**Published:** 2024-08-19

**Authors:** Rumana Islam, Mohammed Tarique

**Affiliations:** 1 Department of Electrical and Computer Engineering, University of Science and Technology of Fujairah (USTF), Fujairah P.O. Box 2202, United Arab Emirates; m.tarique@ustf.ac.ae; 2 Department of Electrical and Computer Engineering, University of Windsor, Windsor, ON N9B 3P4, Canada

**Keywords:** breast cancer, diagnosis, FFNN, FNA, kNN, machine learning, Naïve Bayes, neural network, nuclear features, SVM

## Abstract

Breast cancer is one of the paramount causes of new cancer cases worldwide annually. It is a malignant neoplasm that develops in the breast cells. The early screening of this disease is essential to prevent its metastasis. A mammogram X-ray image is the most common screening tool practiced currently when this disease is suspected; all the breast lesions identified are not malignant. The invasive fine needle aspiration (FNA) of a breast mass sample is the secondary screening tool to clinically examine cancerous lesions. The visual image analysis of the stained aspirated sample imposes a challenge for the cytologist to identify the malignant cells accurately. The formulation of an artificial intelligence-based objective technique on top of the introspective assessment is essential to avoid misdiagnosis. This paper addresses several artificial intelligence (AI)-based techniques to diagnose breast cancer from the nuclear features of FNA samples. The Wisconsin Breast Cancer dataset (WBCD) from the UCI machine learning repository is applied for this investigation. Significant statistical parameters are measured to evaluate the performance of the proposed techniques. The best detection accuracy of 98.10% is achieved with a two-layer feed-forward neural network (FFNN). Finally, the developed algorithm’s performance is compared with some state-of-the-art works in the literature.

## 1. Introduction

**Figure 1:**
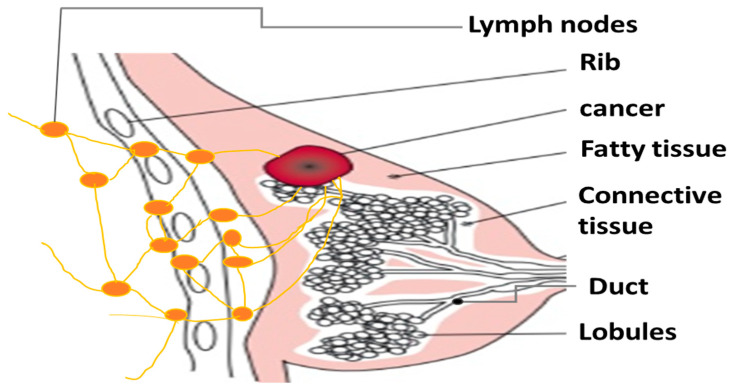
The anatomy and progression of a particular breast cancer.

Breast cancer is one of the most reported invasive cancers. According to recent statistics [1], this disease accounts for 30% of new cancer cases for females in the United States. Breast cancer occurs when some of the breast cells start to grow abnormally. These cells divide themselves more rapidly than healthy ones. Eventually, they may form a lump or a mass identified as a tumor. There are four basic types of breast tumors: benign, normal, carcinoma in situ, and invasive carcinoma [2,3]. A benign one only poses some anatomical changes. Carcinoma in situ is a localized phenomenon that usually refers to the precancerous cells within the boundary of the breast cells from which they originated. Invasive breast cancer usually starts in the breast ducts or glands and develops in breast tissue. It can spread to nearby lymph nodes and other organs if left untreated, as shown in Figure 1 [4].

Currently, an accurate prognosis of breast tumors relies on three systematic approaches: clinical examination, image analysis through a mammogram, and an invasive pathological investigation of the fine needle aspiration cytology (FNAC) sample. The diagnostics of this aspirated image sample is challenging, as experienced by the cytologists [5]. They observe the properties and morphologies of the FNAC sample under a microscopic view. Unfortunately, the benign and malignant (cancerous) cytological samples may have morphological overlaps due to suboptimal sampling techniques or the poor localization of mass lesions [6]. The advanced digital analysis of the cytological image is crucial to support qualitative assessment through the objective and quantitative evaluation of cancerous cells.

Various imaging techniques have been investigated so far for the diagnosis of diseases including breast tumors [7,8,9,10]. These include X-rays, computed tomography (CT) scans, ultrasounds, mammograms, and spectral images. Ultrasounds do not have radiation exposure, as high-frequency sound waves are employed to map the images. However, they are good for prenatal care but unsuitable for disease diagnosis involving bony structures. CT scans can visualize bony structures, soft tissue, and blood vessels at the same time. They can produce high-definition 3D images of a target area. Magnetic resonance imaging (MRI) is often suggested to include a larger suspected region with a more precise scan than a CT scan. MRI is the most expensive option, even though it is free from radiation exposure. X-ray is the cheapest and least time-consuming option compared to ultrasound, CT scan, and MRI. The mammogram is the X-ray image of the suspected breast lesions, as suggested primarily. However, it has less sensitivity in the case of dense breast tissues missing the small tumor [3]. Minimal invasive preoperative screening involves the investigation of cytological images.

Previously, clinicians and researchers have attempted to detect breast cancers by examining the cancerous cells. Through cell nuclei analysis, they have tried to identify malignancy. The accurate classification of breast masses is essential to offer early treatment as the substantive cure for this disease is yet to be discovered.

With the emergence of AI, researchers are actively trying to improve the diagnostic accuracy of malignant breast lesions. In a study [11], George Y. M. et al. investigated four classification models; namely, multilayer perceptron using a back-propagation algorithm, a probabilistic neural network (PNN), learning vector quantization, and a support vector machine (SVM) to identify breast cancer from cytological images. Circular Hough transform, Otsu’s thresholding algorithm, and fuzzy c-means clustering techniques were used to locate the abnormal cell nuclei from the cytological images. PNN and SVM performed well in identifying malignant nuclei from 92 breast cytological images.

Ara, S. et al. developed several machine learning-based algorithms [12] to detect breast cancer from nuclear features of FNAC samples. The best detection accuracy was 96.5% using the Wisconsin Breast Cancer Diagnostic (WDBC) dataset. Also, several machine learning-based algorithms were examined to identify malignant breast cells in [13,14,15] for comparative judgment on detection performance. Reza, A. et al. achieved a significantly high classification accuracy of 99.35% [16] by designing a novel DeepBreastCancerNet deep learning (DL) model from noninvasive ultrasound images of breast lesions. Their proposed model comprised 24 layers, including the convolutional neural networks (CNNs) and inception modules. Reshan, M. S. A. et al. proposed an automated breast cancer prediction model using multi-model features and ensemble machine learning (EML) techniques from FNAC features [17]. They considered the most significant feature of the WDBC dataset to experiment with their model. The best average accuracy was 99.89%.

Singh, S. P. et al. proposed a novel computer-aided system (CAD) [18] to identify breast malignancies from mammographic images. The suspicious region-based polar complex exponential transform (PCET) moments, being texture descriptors, were used as discriminative features. The detection accuracy for malignancy was 97.965%. In another study [19], Guo, R. et al. concluded that breast ultrasound imaging can be helpful and informative in identifying malignancy, even at the lymph nodes in the axilla, between the pectoral muscles, the subclavian region, the neck, and the medial thoracic chain. However, integrating quantitative CAD-based features and correlating them with pathological markers are essential for the best prognosis, as they suggested. Byra, M. et al. proposed a deep learning-based selective kernel (SK) U-Net convolutional neural network [20] to segment the breast mass effectively from the ultrasound images. Their proposed method outperformed conventional U-Net in terms of statistical performance measures.

Togacar, M. et al. developed a CNN-based deep learning model [21] to identify malignant breast samples from histopathological images. The BreastNet model they proposed is a residual architecture built on attention modules. The achieved classification accuracy was 98.8%. A novel deep learning-based network for detecting and classifying breast cancer from cytological images was proposed by Khan, S. et al. in [22]. They considered three transfer learning approaches, GoogleNet, VGG, and ResNet, for their investigation. In transfer learning, the knowledge of related problems is used to solve the investigated problem with a small dataset. The detection and classification accuracy were significant in their work. Nahid, A. et al. designed a combination of CNN and Long-Short-Term-Memory (LSTM) to classify breast masses from histopathological images [23]. An unsupervised clustering operation was performed to extract hidden statistical patterns of the histopathological images; a CNN-based algorithm provided the best detection accuracy.

A noninvasive breast cancer detection method using a low-frequency bioimpedance device was proposed by Mansouri, S. et al. [24]. A measured lower resistance can indicate malignancy in the breast tissues. The device was designed following the Frick’s model. Prasad, A. et al. [25] proposed another noninvasive breast cancer detection system using a Fiber Bragg Grating (FBG) thermal sensor array. A temperature variation of ≥0.3 °C was noted for breast tumors. As the cancer cells have high metabolic activity, this method effectively identified malignant breast lesions at their early stage. The prototype was modeled using COMSOL Multiphysics software.

Ertosun, M. G. et al. proposed a deep learning based approach to search and localize breast mass in mammogram images [26]. Their developed system had two modules for detection and localization of breast mass respectively. The detection accuracy was 85%. Kumar, P. et al. examined an improved CNN-based model to accurately identify breast masses [27] from mammographic images. The detection accuracy was 97.2%. Gupta, K. G. et al. designed a novel lightweight deep learning-based model, ReducedFireNet, to identify breast cancer from histopathological images of breast tissue samples [28]. The mean accuracy was 96.88%. The lightweight design of the proposed system was suitable for the Internet of Medical Things (IoMT) imaging equipment, as the authors claimed. Wang, Z. et al. investigated a combination of deep, morphological, texture, and density features based on mammogram images [29] to detect malignant masses. The developed system worked in two steps; CNN deep features and unsupervised extreme learning machine (ELM) clustering were adopted to identify the masses first, and the feature set was used to detect malignant masses with the designed ELM-based algorithm.

Saidin, N.A. et al. developed a graph cuts algorithm using mammography images for variable breast densities [30]. The quantitative evaluation of breast masses considering breast densities was beneficial for diagnostics. The segmentation of the mammogram into different mammographic densities seemed effective for the risk assessment of breast cancer. Some researchers even correlated the emotional contents of the voice signal to identify the stages of breast cancer [31].

Until now, the image analysis of FNAC samples is being considered the less invasive preoperative screening tool to unveil the malignancy of breast lesions. However, some breast lesions pose additional challenges to characterize malignant cells’ morphology clinically. These may include fibroepithelial lesions, fibrocystic disease, papillary lesions, radial scars and sclerosing adenosis, flat epithelial atypia, borderline proliferative lesions, low-grade carcinoma, etc. [32]. The nature of the lesions also plays a role in the inadequacy of aspirated samples, which is responsible for misdiagnosis [33]. Diagnostic errors can result from an overload of cases and miscorrelation with the patients’ clinical and radiologic findings [34]. Even an experienced cytopathologist cannot reduce the false positive rate in diagnosis.

Researchers have recently been adopting combinational approaches to rule out these limitations, including AI-based techniques. However, the ensemble methods can have additional challenges as they are computationally expensive and time-consuming due to the need to handle multiple models. Also, in having too many layers, these systems’ complexity and memory requirements impose additional issues in interpreting the logic behind the predictions. Considering these challenges, this research focuses on devising a simple network examining discriminative nuclear features to aid breast cancer diagnostics. The significant contributions of this work are (i) the analysis of the essential neural features from the FNAC samples, (ii) the design of an ANN-based optimal FFNN model to identify malignant breast samples, (iii) a performance analysis of the proposed model with significant statistical measures, and (iv) a comparison of the devised method with some state-of-the-art work in the literature.

The remainder of this research is structured as follows: Section 2 describes the materials and methods and Section 3 represents the classification results, including the comparison. Section 4 constitutes discussions. Finally, Section 5 concludes the proposed research with future directions.

## 2. Materials and Methods

### 2.1. The Data Samples and Features

This investigation employs the publicly available WBDC dataset from the UCI repository [35]. The developer of this dataset is Dr. William H. Wolberg, University of Wisconsin Hospital in Madison, Wisconsin, USA. This database comprises 569 samples, each having 30 discriminative real-valued nuclear features. Among the 569 samples, 357 are benign or non-cancerous and 212 are malignant. The nuclear features were computed from the digitized image of the FNAC samples. The cell features used in this work are the actual boundary of the cell nucleus located by an active contour model known as a “snake”. A snake minimizes an energy function defined over the arc of a closed curve. The energy function is defined in such a way that the minimum value occurs when the curve accurately corresponds to the boundary of a cell nucleus [36]. The discriminative ten (10) nuclear features for malignant and healthy breast tissue samples are defined in Table 1. The mean, standard error (se), and worst (mean of the three largest values) of these features were computed for each image, resulting in 30 features. A full explanation of the estimation techniques of these features can be found in [36].

**Figure 2:**
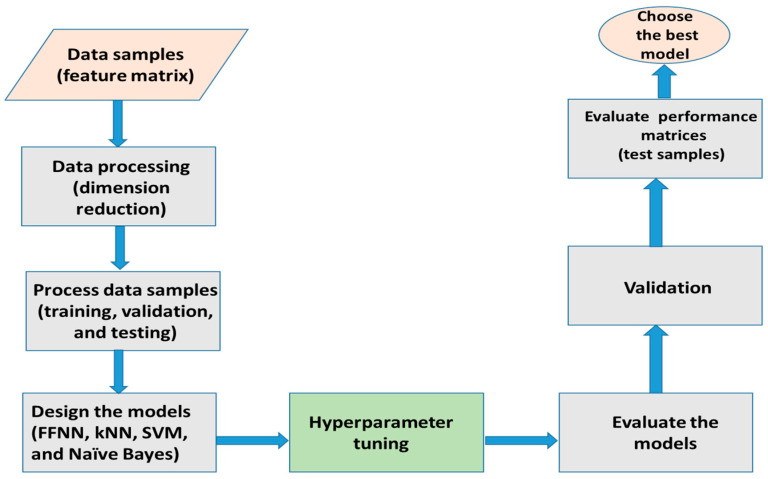
The proposed methodology.

**Figure 3:**
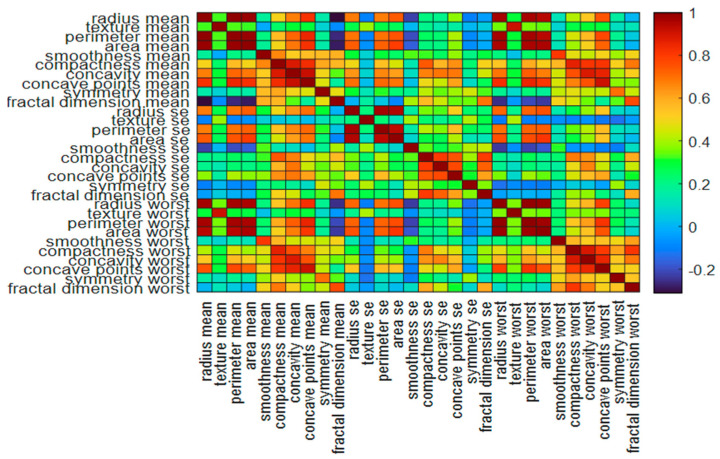
The heatmap of the thirty (30) nuclear features.

The flow diagram for the proposed methodology is shown in Figure 2. The heatmap of the 30 nuclear features is shown in Figure 3. Some features are highly correlated with each other compared to others, as demonstrated by the intensity of color portrayed in the colorbar. For example, the perimeter, area, compactness, concavity, and concave points depicted a higher correlation. So, the feature dimension is reduced considering the 95% variance using principal component analysis [38] to avoid overfitting when designing an automated classification network.

### 2.2. The Classification Network

**Figure 4:**
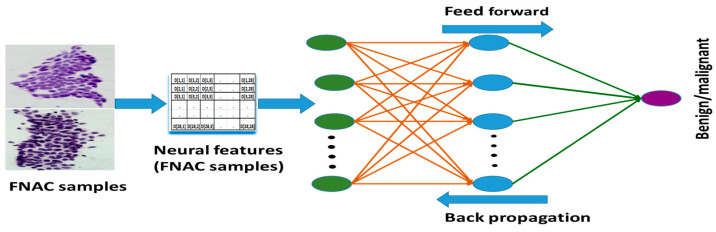
The system model for FFNN-based classification network.

An artificial two-layer neural network-based feed-forward neural network (FFNN) is deployed for this research to detect malignancies from the FNAC samples. The system model is shown in Figure 4.

The nuclear features extracted from the digitized sample images are fed to the classification network. This network consists of neurons ordered into layers. The first layer is the input layer, the last layer is the output layer, and the layers in between are the hidden layers. The interconnections between the neurons are weighted based on the importance of connections between the nodes. The FFNN is trained by using the scaled conjugate gradient backpropagation algorithm. This algorithm utilizes the gradient descent technique to reduce the cost function. The cost function, which the backpropagation network tries to minimize, is the squared difference between the actual network output and the target or desired output value summed over all the output units.

As mentioned earlier, the input is the feature matrix of 569 samples (357 are benign and 212 are malignant) with 30 attributes. The optimum number of neurons in the hidden layer was 10 for the input feature matrix. The transfer function used for the hidden layer is the Sigmoid function, and for the output neuron it is SoftMax function. The data samples are divided into three parts. Seventy percent (70%) of the data is used for training. The remaining 30% of the data is equally divided for validation and testing. There is only one output node as the decision is binary (i.e., malignant or benign).

## 3. Results

The proposed system is evaluated with the following parameters: (a) true positive ($t_{p}$), (b) true negative ($t_{n}$), (c) false positive ($f_{p}$), and (d) false negative ($f_{n}$). Also, the subsequent performance measures, defined by *t_p_*, *t_n_*, *f_p_*, and *f_n_* that address the results of binary classification are as follows [39]:

(1) $$accuracy=\frac{t_{p}+t_{n}}{t_{p}+t_{n}+f_{p}+f_{n}}$$

(2) $$precision=\frac{t_{p}}{t_{p}+f_{p}}$$

(3) $$recall=\frac{t_{p}}{t_{p}+f_{n}}$$

(4) $$negative\;predictive\;value,\;npv=\frac{t_{n}}{t_{n}+f_{n}}$$

(5) $$F1\;Score=\frac{2*recall*precision}{recall+precision}$$

(6) $$specificity=\frac{t_{n}}{t_{n}+f_{p}}$$

(7) $$false\;negative\;rate,\;fnr=\frac{f_{n}}{f_{n}+t_{p}}$$

(8) $$false\;detection\;rate,\;fdr=\frac{f_{p}}{f_{p}+t_{p}}$$

(9) $$G–mean=\sqrt{sensitivity*specificity}$$

(10) $$\mathrm{Matthew}’s\mathrm{Correlation}\mathrm{Coefficient},\;MCC=\frac{t_{p}*t_{n}-f_{p}*f_{n}}{\sqrt{{(t}_{p}+f_{p}){(t}_{p}+f_{n}){(t}_{n}+f_{p}){(t}_{n}+f_{n})}}$$

(11) $$\mathrm{Dice}\mathrm{Score},\;DSc=\frac{2t_{p}}{{2t}_{p}+f_{p}+f_{n}}$$

The performance measures of the proposed algorithm are listed in Table 2, considering the overall performances of training, validation, and testing. This table shows that the proposed algorithm achieves an overall *accuracy* of 98.10%. The other performance metrics, *precision*, *recall*, and *f*_1_-*score*, are 98.60%, 96.20%, and 97.40%, respectively. The remaining other measures, namely *npv*, *specificity*, *fnr*, *fdr*, *G-mean*, *MCC*, and *DSc*, are 97.80%, 99.20%, 1.45%, 2.21%, 97.70%, 95.90%, and 97.40%, respectively. The *MCC* representing the confusion matrix with a single parameter (i.e., 95.90%) is reasonably satisfactory. The *G*-*mean*, which identifies the balance between the majority and minority classes, is 97.70%, resembling an excellent performance. The *DSc* of 97.40% indicates that the results are nearly identical to the ground truth.

**Figure 5:**
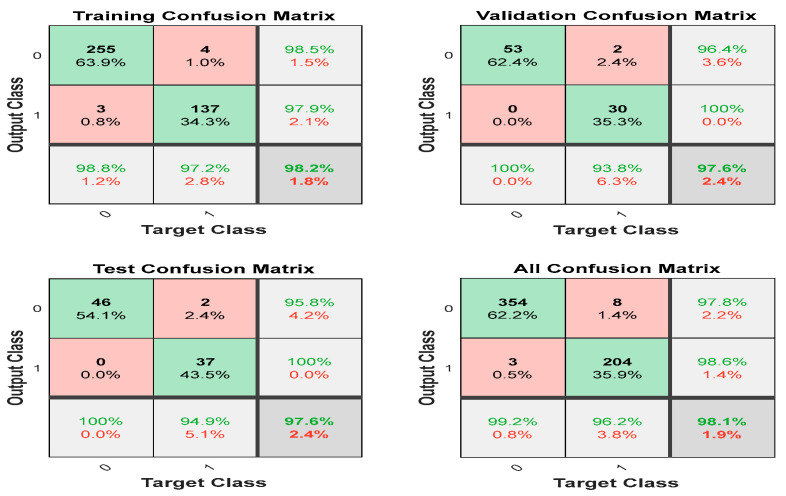
The confusion matrices for training, validation, testing, and overall cases; 0 indicates benign, and 1 indicates malignancy.

The corresponding confusion matrices are shown in Figure 5. As mentioned above, 70% of the data samples (i.e., 399) were used for the training. The confusion matrix for the training is shown in Figure 5 in the top left corner. It indicates that the *t_p_* and *t_n_* were 137 and 255, respectively. The *f_p_* and *f_n_* are only 3 and 4, respectively. These values indicate unbiased training. The confusion matrix for the validation and testing also demonstrates similar unbiasedness. The combined confusion matrix is also presented in the same figure at the bottom right corner. The green diagonal elements are percentages of correctly classified cases. The corresponding off-diagonal elements are percentages of misclassified cases. It also shows that the model accurately detects 255 benign and 137 malignant trained samples. The bottom right cell for all confusion matrices indicates the overall correctly predicted classes (in green %), that is 98.10%, considering training, testing, and validation performance. It also displays the overall misclassified cases, i.e., 1.9% (in red).

**Figure 6:**
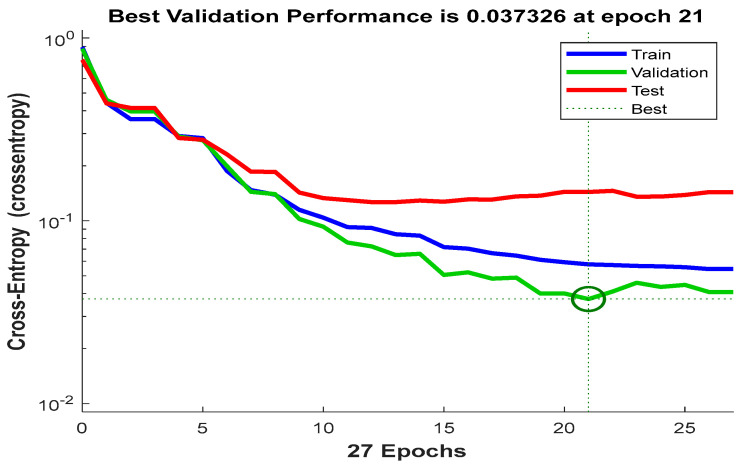
The cross-entropy function for training, testing, and validation data.

The cross-entropy function of the training, validation, and testing samples are plotted in Figure 6. It displays the cross-entropy loss between the predictions and targets. This figure shows that the minimum best validation cross-entropy is 0.037326, which was achieved at epoch 21. No significant changes occurred after epoch 21. The training stopped at epoch 27 after six (6) iterations of the best validation point (i.e., epoch 21). The system performance is significantly high since the magnitude of the final cross-entropy is insignificant. The validation and test cross entropy have almost similar patterns. No significant overfitting happened before epoch 21, where the best validation occurred.

**Figure 7:**
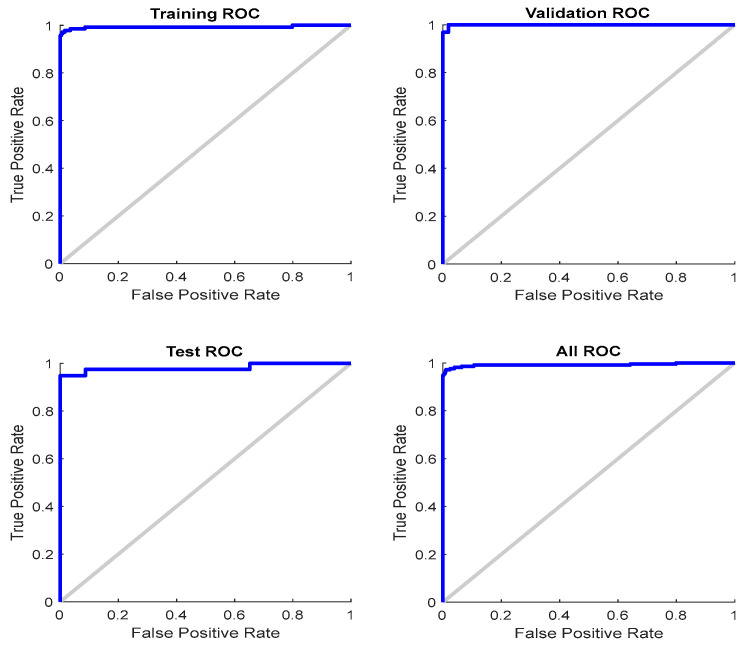
The ROC curves for training, validation, testing, and overall cases.

The ROC curve of training, validation, and testing are presented in Figure 7. They are displayed as plots of the true positive rate (i.e., sensitivity) vs. the false positive rate (i.e., specificity) with the variation of threshold values. The perfect test would show the points on the upper left corner resembling 100% sensitivity and 100% specificity. Considering all ROCs (training, validation, and testing), this system performs reasonably well.

## 4. Discussion

**Figure 8:**
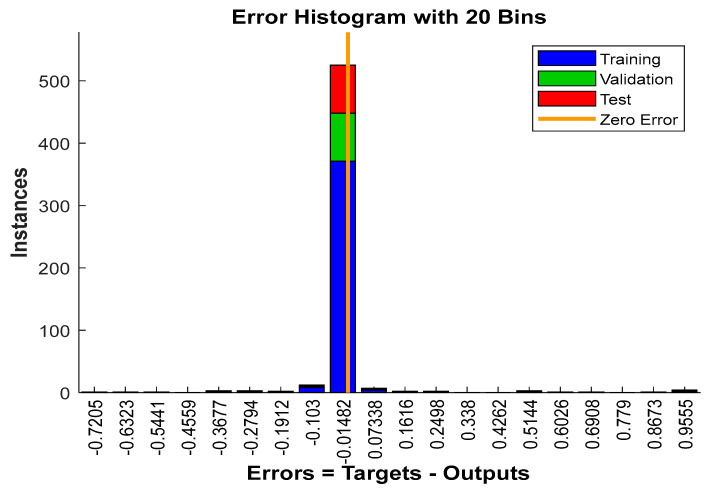
The error histogram plot of the system model for 20 bins.

The error histogram plot of the proposed model for 20 bins is shown in Figure 8. This system model significantly anticipated zero error for the ninth bin. The ninth bin constitutes the major components of training, validation, and testing data samples.

To compare the achieved results, several machine learning-based algorithms were also investigated to identify breast cancers from the same WDBC data samples. The best detection performance measures are obtained for Cubic SVM, as shown in Table 3, considering the 5-fold cross validation scheme. Comparing Table 2 and Table 3, it can be concluded that the overall best detection performance is achieved with the FFNN algorithm.

**Figure 9:**
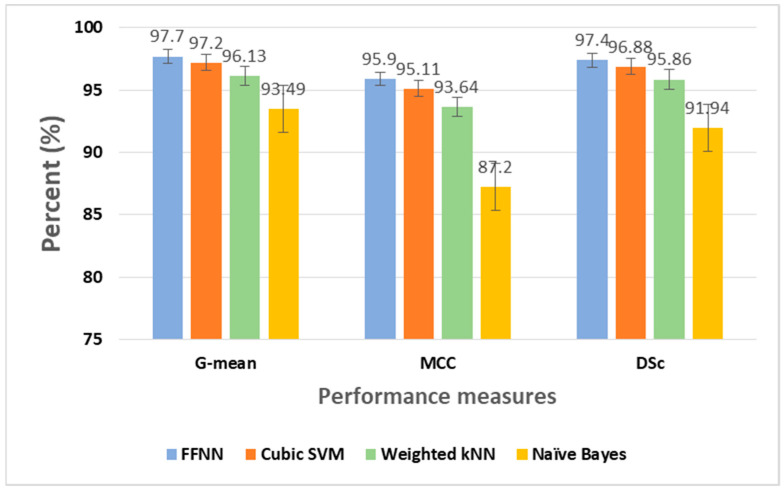
A performance analysis of the developed algorithms.

A comparative visual analysis of the obtained results considering significant performance measures and error bars is shown in Figure 9. All the classification networks were modeled with MATLAB 2020 software.

Finally, a performance comparison of this research is presented with some state-of-the-art works in the literature, as shown in Table 4. The proposed system achieved better accuracy than the works in [11,12,13] using the FNAC samples. The works in [15,17] achieved better accuracy considering the morphological features from FNAC samples. The work in [17] was designed with an ensemble machine learning algorithm, considering a multitude of classifiers. Also, significantly high accuracy was achieved in [16,21] from the ultrasound and histopathological images, respectively.

This research to devise a breast cancer detection algorithm is promising. It suggests an intriguing method for managing suspicious breast lesions; it may be more applicable as a screening tool rather than a definitive diagnostic method. However, even though FNAC is a fast, economical, less invasive preoperative procedure, as per National Comprehensive Cancer Network (NCCN) guidelines [40], the gold standard for diagnostics is the ultrasound guided core needle biopsy. This research methodology can also be applicable to image samples collected from core needle biopsies (CNB), and the surgical open biopsies (SOB) provided the significant features could be extracted and researched following statistical analysis and using digital technology.

## 5. Conclusions

Breast cancer is one of the most common cancers, taking an enormous number of precious lives worldwide. But early detection could lower the mortality rate, saving many lives. AI is in an appreciably predominant position to aid disease diagnosis currently. With the advent of data mining, it has led researchers to additional possibilities to explore the methods of early screening to prevent cancer recurrence.

Despite the continuous development of computational cytology in recent years, there are still challenges and open problems in precisely identifying the malignant neoplasms of the breast cells. So, advanced techniques to handle the poor localization of malignant cells are essential. This research presented several automated breast cancer detection algorithms based on nuclear features extracted from the FNAC samples. However, the FFNN algorithm achieved the best results. The discriminative power of multidimensional nuclear features, setting an optimum number of neurons in the hidden layers, enabled the simple and shallow network to perform well. The computational burden of the generated system is significantly low. This study will contribute to the early screening and clinical prognosis of breast cancer patients.

However, the proposed research only considers the binary detection of malignant lesions and healthy breast cells. The multiclassification of FNAC samples to identify the stages of malignancy from suspicious lesions is left for future investigation. Domain-specific feature analysis to correlate the clinical results needs particular attention as well. Also, in the future, the proposed algorithm will be experimented on further regarding histological breast samples obtained from core needle biopsies to enhance diagnostic success.

## Figures and Tables

**Table 1:** The description of nuclear features [36].

Features	Definition	Malignant Mean (Mean, se, and Worst)	Benign Mean (Mean, se, and Worst)
Radius	Radius is measured by averaging the length of the radial line segments defined by the centroid of the snake and the individual snake points.	(17.46, 0.6, 21.13)	(13.61, 0.37, 15.48)
Texture	The texture of the cell nucleus is measured by finding the standard deviation or variance of the gray scale intensities in the component pixels of each image.	(21.60, 1.21, 29.31)	(19.14, 1.24, 25.37)
Perimeter	The total distance between the snake points constitutes the nuclear perimeter.	(115.36, 4.32, 141.37)	(88.35, 2.64, 101.72)
Nuclear area	Nuclear area is measured by counting the number of pixels on the interior of the snake and adding one-half of the pixels in the perimeter.	(978.37, 72.67, 1422.29)	(606.38, 35.3, 795.16)
Smoothness	Smoothness of a nuclear contour is quantified by measuring the difference between the length of a radial line and the mean length of the lines surrounding it.	(0.10, 0.0068, 0.145)	(0.095, 0.007, 0.123)
Compactness	Compactness is defined by combining the perimeter and area to give a measure of the compactness of the cell nuclei using the formula: perimeter^2^/area.	(0.015, 0.032, 0.375)	(0.097, 0.024, 0.23)
Concavity	Concavity is defined by drawing chords between non-adjacent snake points and measuring the extent to which the actual boundary of the nucleus lies on the inside of each chord.	(0.16, 0.042,0.45)	(0.078, 0.031, 0.024)
Concave points	Concave points are like the concavity but measure only the number, rather than the magnitude of contour concavities.	(0.088, 0.015, 0.182)	(0.042, 0.011, 0.012)
Symmetry	The symmetry is computed by measuring the major axis through the center and then measuring the length difference between lines perpendicular to the major axis to the cell boundary in both directions.	(0.190, 02, 0.323)	(0.18, 0.02, 0.284)
Fractal Dimension	The fractal dimension of a cell is approximated using the “coastline approximation-1” described by Mandelbrot [37].	(0.062, 0.004, 0.09)	(0.062, 0.0038, 0.082)

**Table 2:** The performance measures of the FFNN algorithm (considering training, validation, and testing performances).

Performance Measures	(%)
*accuracy*	98.10 ± 1.01
*precision*	98.60 ± 1.01
*recall*/*sensitivity*	96.20 ± 1.02
*F*1 *Score*	97.40 ± 1.03
*npv*	97.80 ± 1.02
*specificity*	99.20 ± 1.02
*fnr*	1.45 ± 0.02
*fdr*	2.21 ± 0.01
*G*-*mean*	97.70 ± 1.01
*MCC*	95.90 ± 1.02
*DSc*	97.40 ± 1.03

**Table 3:** The performance measures of the machine learning algorithms. (Considering 5-fold cross validation).

Performance Measures	Cubic SVM (%)	Weighted kNN (%)	Gaussian Naive Bayes (%)
*accuracy*	97.72 ± 1.03	97.01 ± 1.13	94.02 ± 1.01
*precision*	98.54 ± 1.01	98.99 ± 1.14	92.38 ± 1.02
*recall*/*sensitivity*	95.28 ± 1.11	92.92 ± 1.12	91.51 ± 1.03
*F*1 *Score*	96.88 ± 1.01	95.86 ± 1.11	91.94 ± 1.03
*npv*	97.25 ± 1.01	95.95 ± 1.11	94.99 ± 1.11
*specificity*	99.16 ± 1.02	99.44 ± 1.12	95.52 ± 1.02
*fnr*	1.46 ± 0.11	1.01 ± 0.03	7.62 ± 0.01
*fdr*	2.75 ± 0.02	4.05 ± 0.02	5.01 ± 0.02
*G*-*mean*	97.20 ± 1.12	96.13 ± 1.13	93.49 ± 1.11
*MCC*	95.11 ± 1.11	93.64 ± 1.11	87.20 ± 1.12
*Dice Score*	96.88 ± 1.13	95.86 ± 1.15	91.94 ± 1.13

**Table 4:** The performance comparison with some state-of-the-art works.

Research Works	Samples	Features	Tools	Best Accuracy (%)
George Y. M. [11]	FNAC	Cell nuclei	Multilayer perceptron, PNN, learning vector quantization (LVQ), and SVM	95.56
Ara, S. [12]	FNAC	Cell nuclei	Random Forest, Logistic Regression, Decision Tree, Naive Bayes, SVM, and kNN	96.50
Khourdifi, Y [13]	FNAC	Cell nuclei	Random Forest, Naïve Bayes, SVM, and kNN	97.90
Islam M. [15]	FNAC	Morphological Features	SVM and kNN	**98.57**
Raza, A. [16]	Ultrasound	Breast lesions	DeepBraestCancerNet	**99.35**
Reshan, MSA [17]	FNAC	Morphological features	Ensemble machine learning	**99.89**
Singh, S. P [18]	Mammographic images	PCET moments	Adaptive Differential Evolution Wavelet Neural Network (ADEWNN)	97.96
Byra, M. [20]	Ultrasound	Segmentation	Selective kernel (SK) U-Net CNN	97.90
Togacar, M. [21]	Histopathological images	Original image	BreastNet	**98.80**
Nahid, A. [23]	Histopathological images	*k*-means and Mean-Shift clustering algorithm	CNN-LSTM	91
Ertosun, M. G [26]	Mammogram	In built feature extractor	CNN	85%
Gupta, K. G [28]	Histopathological images	Image Enhancement	ReducedFireNet	96.88
Wang, Z. [29]	Mammogram	Deep features, morphological features, texture features, density features	CNN and unsupervised Extreme learning machine (ELM)	86.50
**Proposed work**	**FNAC**	**Nuclear features**	**FFNN**, SVM, kNN, and Naïve Bayes	**98.10 (FFNN)**

## Data Availability

Publicly available datasets were analyzed in this study. These data can be found here: https://archive.ics.uci.edu/dataset/17/breast+cancer+wisconsin+diagnostic (accessed on 9 July 2024).
